# Editorial: Stress and reproduction in animal models

**DOI:** 10.3389/fendo.2023.1202275

**Published:** 2023-05-05

**Authors:** Takayoshi Ubuka, Guixian Bu, Yasuko Tobari

**Affiliations:** ^1^ Initiative for Research and Development, International Cancer Laboratory Co., Ltd., Tokyo, Japan; ^2^ College of Life Science, Sichuan Agricultural University, Ya’an, China; ^3^ School of Veterinary Medicine Department of Animal Science and Biotechnology, Azabu University, Sagamihara, Japan

**Keywords:** HPG axis, circRNA, ovulation, oxidative stress, symbiotic combination

It is well known that reproductive functions are suppressed or altered under stress. Environmental factors, infection, malnutrition, and chronic strenuous experience can all lead to alterations in reproductive functionality in animals. Stressors interfere with the timing and regulation of reproductive hormones, such as gonadotropin-releasing hormone (GnRH), gonadotropin-inhibitory hormone (GnIH), gonadotropins and sex steroids and processes within the hypothalamic-pituitary-gonadal (HPG) axis (1–5). While similar in function, these processes and their related hormones can present themselves differently in animal models compared to humans. Understanding these differences is crucial to furthering our understanding of how research with animal models should be applied to humans and how it can advance commercial applications for those animal models. This Research Topic compiles four original research that enhance and expand our understanding of how stress affects reproductive functionality in animal models.

Photoperiod is a key factor for organisms to reproduce seasonally to maximize the survival of their offspring. The thyroid gland plays an important role in organismal development and homeostasis including regulation of seasonal reproduction in seasonally breeding animals (6). In sheep, although thyroidectomy has no clear effect on the transition to reproduction in late summer, it blocks the transition to non-breeding in late winter (7). The first original research in this Research Topic performed a comprehensive analysis of circular RNA (circRNA) profiles in the thyroid gland of ovariectomized (OVX) plus E_2_ treated ewes at short and long photoperiods by whole transcriptome sequencing. CircRNAs are stable biomolecules that have a covalently closed structure and are not degraded by RNase (8). CircRNAs interact with microRNAs, and they can regulate immune responses and behavior (9). However, circRNA profiles in the thyroid gland under different photoperiods were completely unknown. This study detected 37,470 novel circRNAs in different photoperiods. Functional enrichment annotation analysis featured inositol phosphate metabolism, cGMP-PKG, calcium, MAPK signaling pathways, and oocyte meiosis that influence photoperiod responses in sheep. This study also revealed target binding sites for microRNAs in circRNAs by competitive endogenous RNA network analysis. The results of this study provide new information on circRNA function and changes in the thyroid gland under different photoperiods in sheep.

The second paper performed a meta-analysis on 517 papers published in the past 30 years about the effect of androgens on the ovulation rate in sows. Previous studies reported that exogenous testosterone (T) injection improves the ovulation rate in sows (10, 11). However, there is a disagreement about the effect of T injection on the survival rate of embryos in sows (11, 12). There are reports showing that dihydrotestosterone (DHT) injection decreases the survival rate of embryos in sows (11, 13). The meta-analysis showed that both T and DHT injections are positively related to the ovulation rate. However, T did not have a relevant effect on blastocyst survival rate. On the other hand, DHT had a negative phase regarding its effect on blastocyst survival rate. This paper concludes that future research should focus on the mixed use of T and DHT to improve the litter size of sows. The authors also discuss that the timing of their usage should be consistent with the changes in the androgen levels in future research.

The third original research paper aims to explore the mechanism of dysfunction in testosterone production in obese male mice exposed to chronic high-altitude hypoxia. Obesity is frequently characterized by low testosterone level that impairs male fertility, bone mineralization, fat metabolism, and muscle mass (14). Oxidative stress (OS) in the testis is thought to play a critical role in male hypogonadism (15). OS is also thought to be a crucial pathogenic factor in male hypogonadism induced by obesity (16). Hypoxia increases the production and accumulation of reactive oxygen species (ROS) that causes OS (17). Chronic high-altitude hypoxia aggravated low testosterone production in obese male mice. The testis of the mice indicated OS and histological damages. Proteomic analysis in the testis demonstrated that pathways related to testosterone production and function were altered including cholesterol metabolism, steroid hormone production, peroxisome proliferator-activated receptor signaling pathway, as well as OS responses and nitinol metabolism. StAR, DHCR7, NSDHL, CYP51A1, FDPS, FDX1, CYP11A1, ALDH1A1 and GPX3 were downregulated in Obese/Control and Obese-Hypoxia/Obese groups. The authors conclude that chronic hypoxia may exacerbate low testosterone production in obese male mice through key proteins related to cholesterol and steroid hormone biosynthesis, OS responses, and retinol metabolism.

The last original research investigated the effects of a symbiotic combination (Syn) of *Lactobacillus gasseri* 505 (505) and *Cudrania tricuspidata* leaf extract (CT) on the HPG axis of mice under chronic stress. The authors’ previous study in mice showed that a probiotic strain 505 isolated from infant feces and its Syn with CT a newly described plant-based prebiotics improved antioxidative and anti-inflammatory activities and prevented hepatotoxic effects associated with colorectal cancer (18, 19). CT contains a variety of phenolic acids and flavonoids such as predominant neo-chlorogenic acid, chlorogenic acid, caffeic acid, and quercetin-3-glucoside (20). Unpredictable chronic mild stress (UCMS) group mice were exposed to repeated mild physical and psychological stressors including sleep cycle changes, wet bedding, tilted cages, changes in illumination, water deprivation, restraint, and cold-water bath in randomized orders each day, while the control group mice remained undisturbed except housekeeping during the experiment. Syn suppressed UCMS-induced increase in the serum level of corticosterone. Syn also repaired histopathological damage under UCMS. Down regulation in the transcription levels of GnRH, GnRH receptor, gonadotropins, genes related to testicular development and steroidogenesis by UCMS was also inhibited by Syn. The authors conclude that Syn could attenuate testicular dysfunctions induced by UCMS.

The research articles investigated the reproductive activities of sheep, sows and mice focusing on the thyroid gland and the HPG axis. The analyses included the mechanism of how stress suppresses reproductive activities and prevents the negative effect of stress on reproduction. The four articles thus contribute to our deeper understanding of how stress affects reproduction and its prevention in animal models.

## The articles‘ order of this research topic

1. Wei Wang, Xiaoyun He, Ran Di, Xiangyu Wang, Mingxing Chu. Photoperiods induced the circRNA differential expression in the thyroid gland of OVX+E2 ewes. Front Endocrinol (Lausanne) (2022) 13:974518. doi: 10.3389/fendo.2022.974518. eCollection 2022.
2. Zhenhua Guo, Lei Lv, Di Liu, Hong Ma, Cedomir Radovic. A meta-analysis: Effect of androgens on reproduction in sows. Front Endocrinol (Lausanne) (2023) 14:1094466. doi: 10.3389/fendo.2023.1094466. eCollection 2023.
3. Shuqiong Wang, Youwen Wei, Caiyan Hu, Fang Liu. Proteomic analysis reveals proteins and pathways associated with declined testosterone production in male obese mice after chronic high-altitude exposure. Front Endocrinol (Lausanne) (2022) 13:1046901. doi: 10.3389/fendo.2022.1046901. eCollection 2022.
4. Jae Yeon Joung, Whasun Lim, Yeon Jeong Seo, Jiyeon Ham, Nam Su Oh, Sae Hun Kim. A Synbiotic Combination of Lactobacillus gasseri 505 and Cudrania tricuspidata Leaf Extract Prevents Stress-Induced Testicular Dysfunction in Mice. Front Endocrinol (Lausanne) (2022) 13:835033. doi: 10.3389/fendo.2022.835033. eCollection 2022.

## Author contributions

TU wrote the manuscript. GB and YT read the manuscript. All authors contributed to the article and approved the submitted version.
